# Supplementary material for: Quantification of Protein Uptake by Endocytosis in Carnivorous Nepenthales
Source: Plants (Basel). 2023 Jan 11;12(2):341. doi: 10.3390/plants12020341 (PMC9863405; doi:10.3390/plants12020341)
Supplement: Supplementary file 1 [file plants-12-00341-s001.zip › plants-2107158-supplementary.pdf]

plants MDPI

Supplementary Materials:

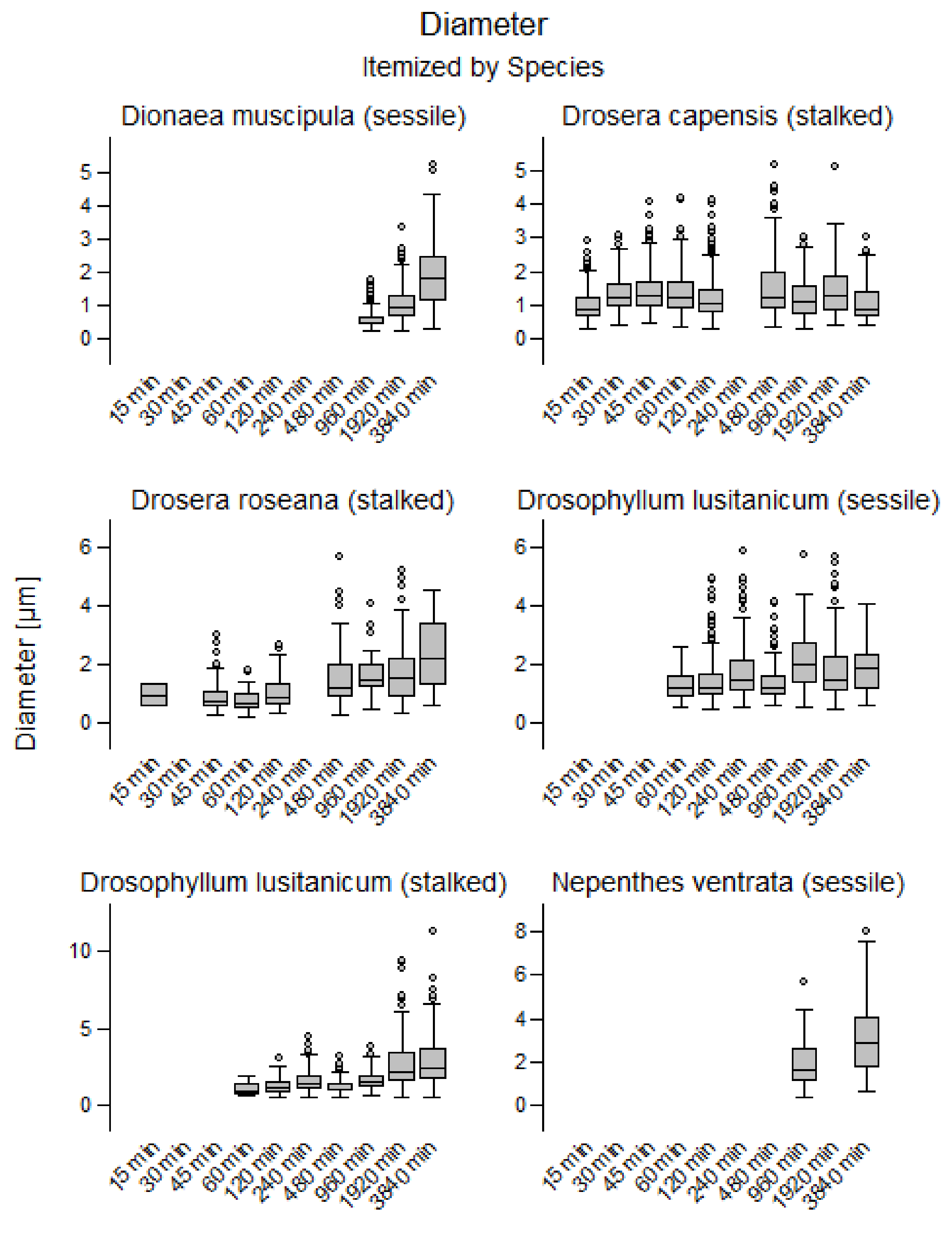

Figure S1. Boxplots of vesicle diameters in observed glandular cells in *Dionaea muscipula*, *Drosera roseana*, *Drosera capensis*, *Nepenthes × ventrata* and stalked and sessile glands in *Drosophyllum lusitanicum*.

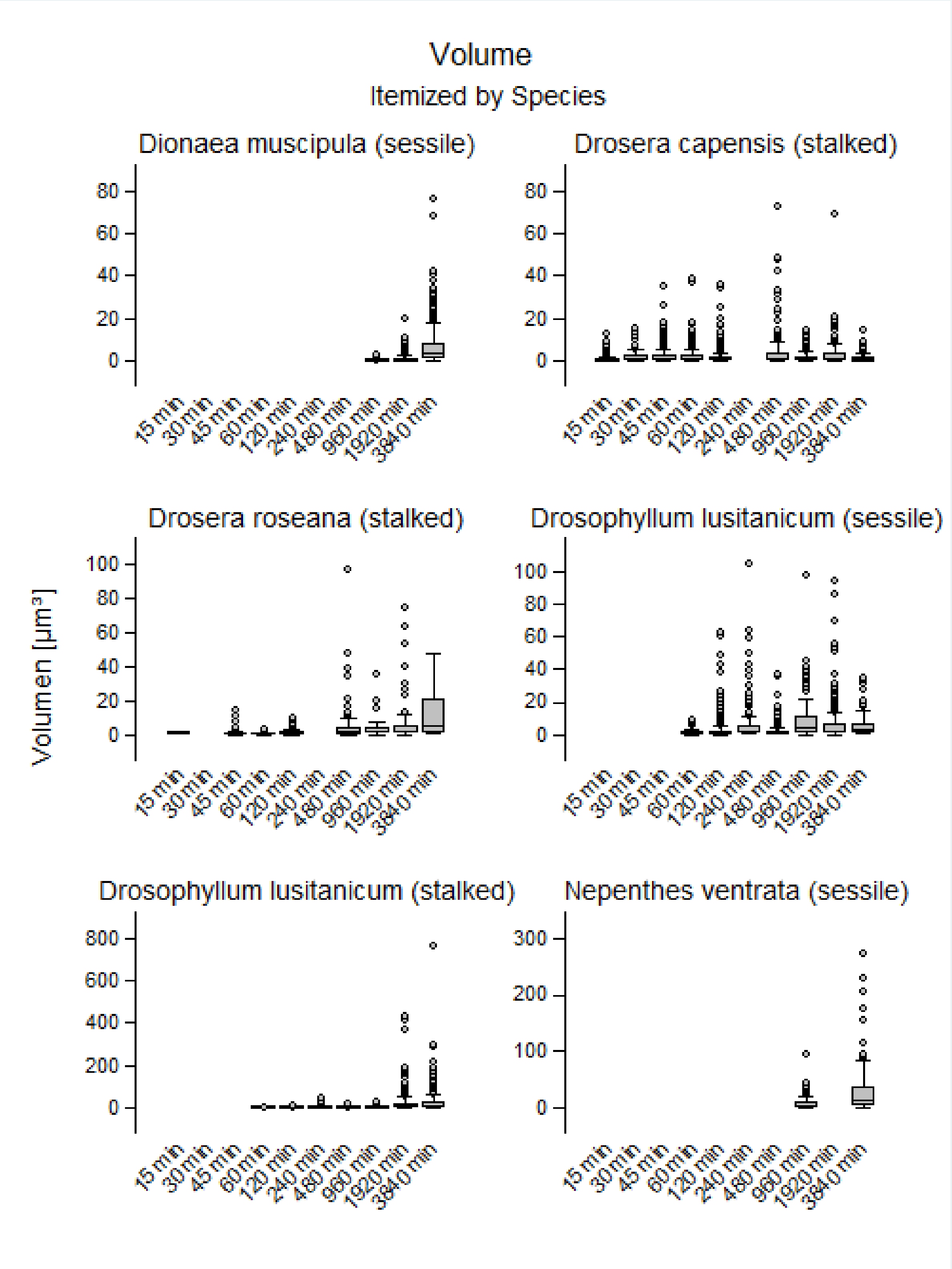

Figure S2. Boxplot of vesicle volumes in observed glandular cells in *Dionaea muscipula*, *Drosera roseana*, *Drosera capensis*, *Nepenthes × ventrata* and stalked and sessile glands in *Drosophyllum lusitanicum*.

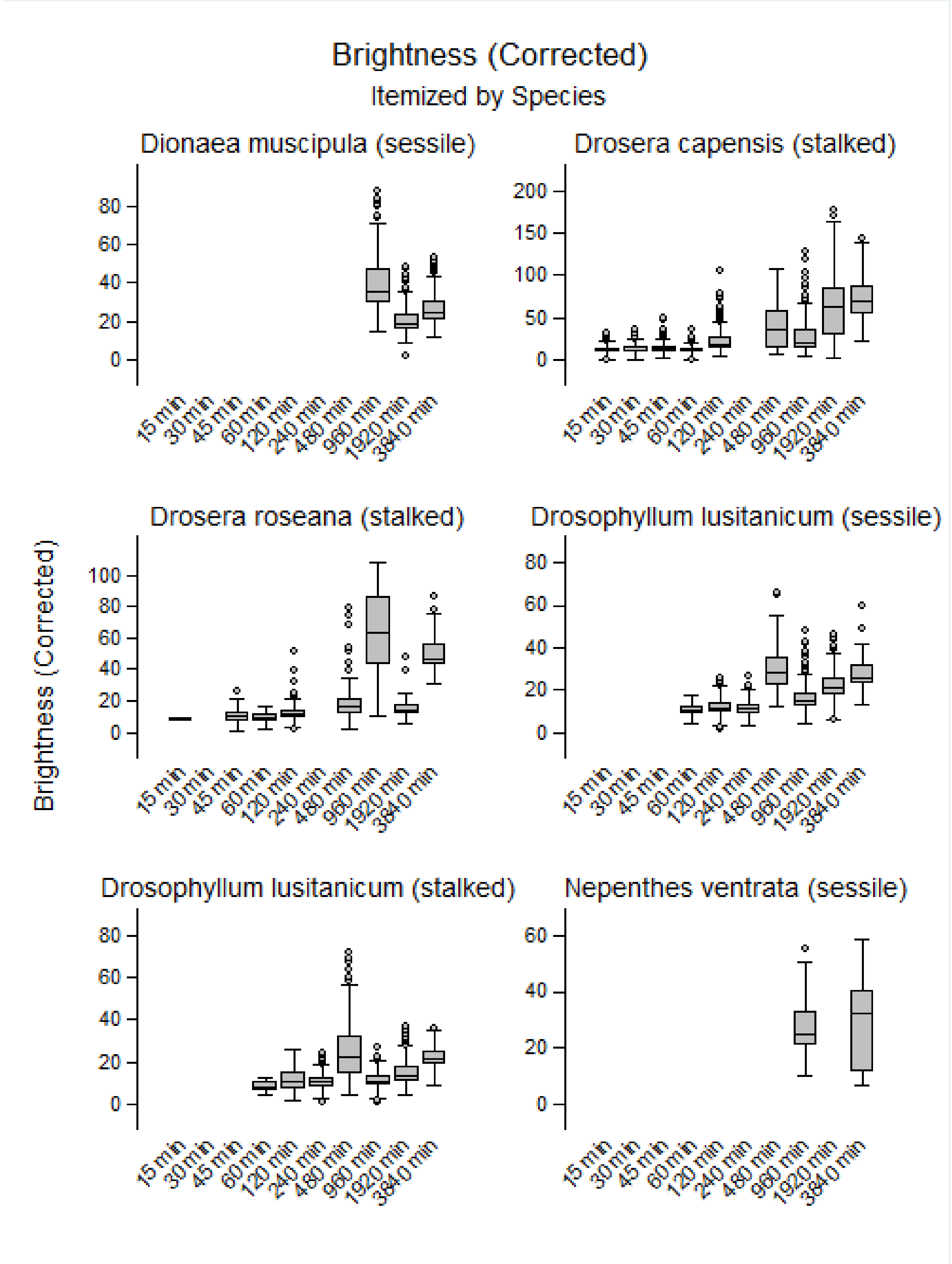

Figure S3. Boxplot of corrected brightness of vesicles in observed glandular cells in *Dionaea muscipula*, *Drosera roseana*, *Drosera capensis*, *Nepenthes × ventrata* and stalked and sessile glands in *Drosophyllum lusitanicum*.

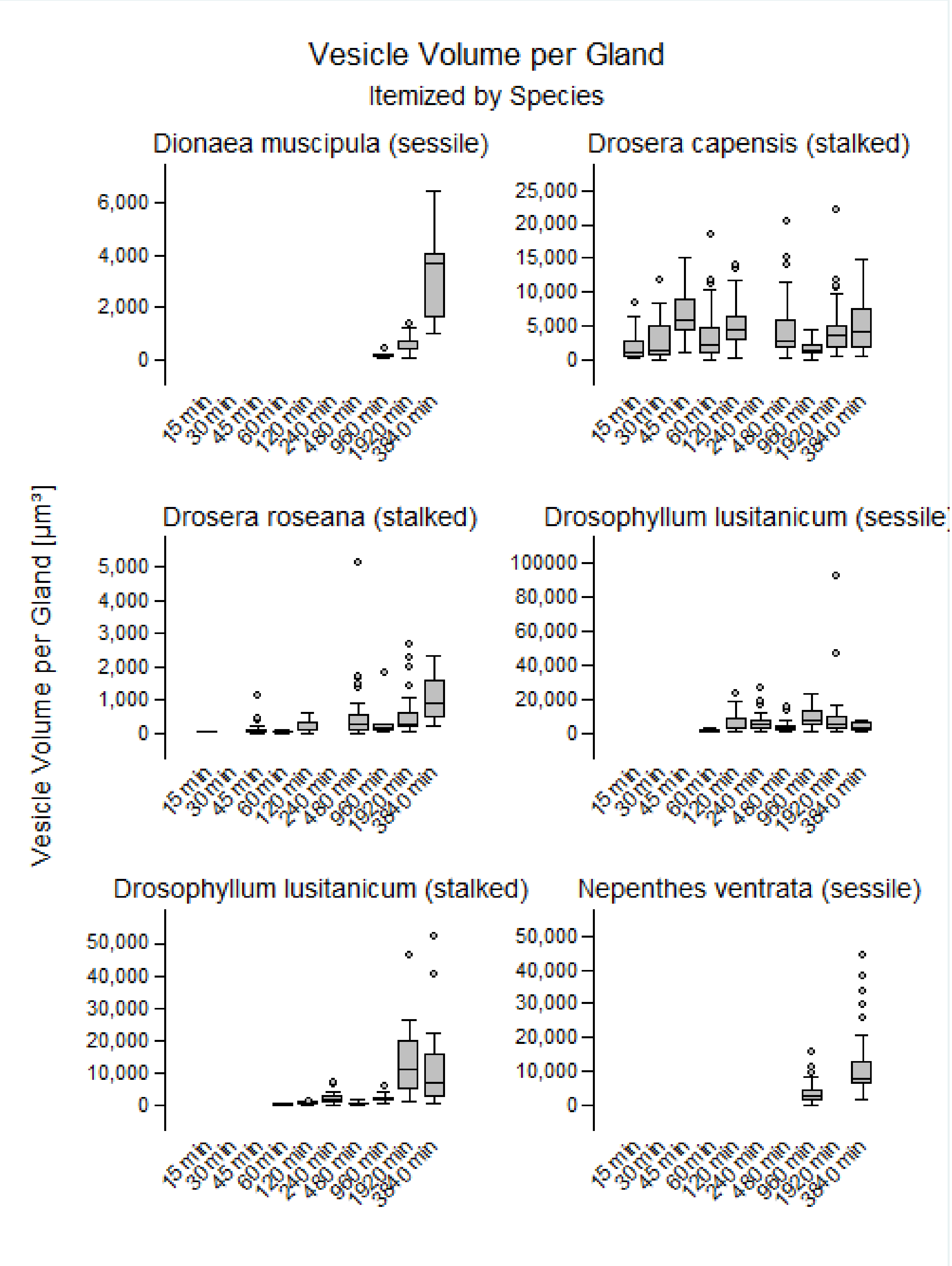

Figure S4. Boxplots of vesicle volumes in observed glands in *Dionaea muscipula*, *Drosera roseana*, *Drosera capensis*, *Nepenthes* × *ventrata* and stalked and sessile glands in *Drosophyllum lusitanicum*.

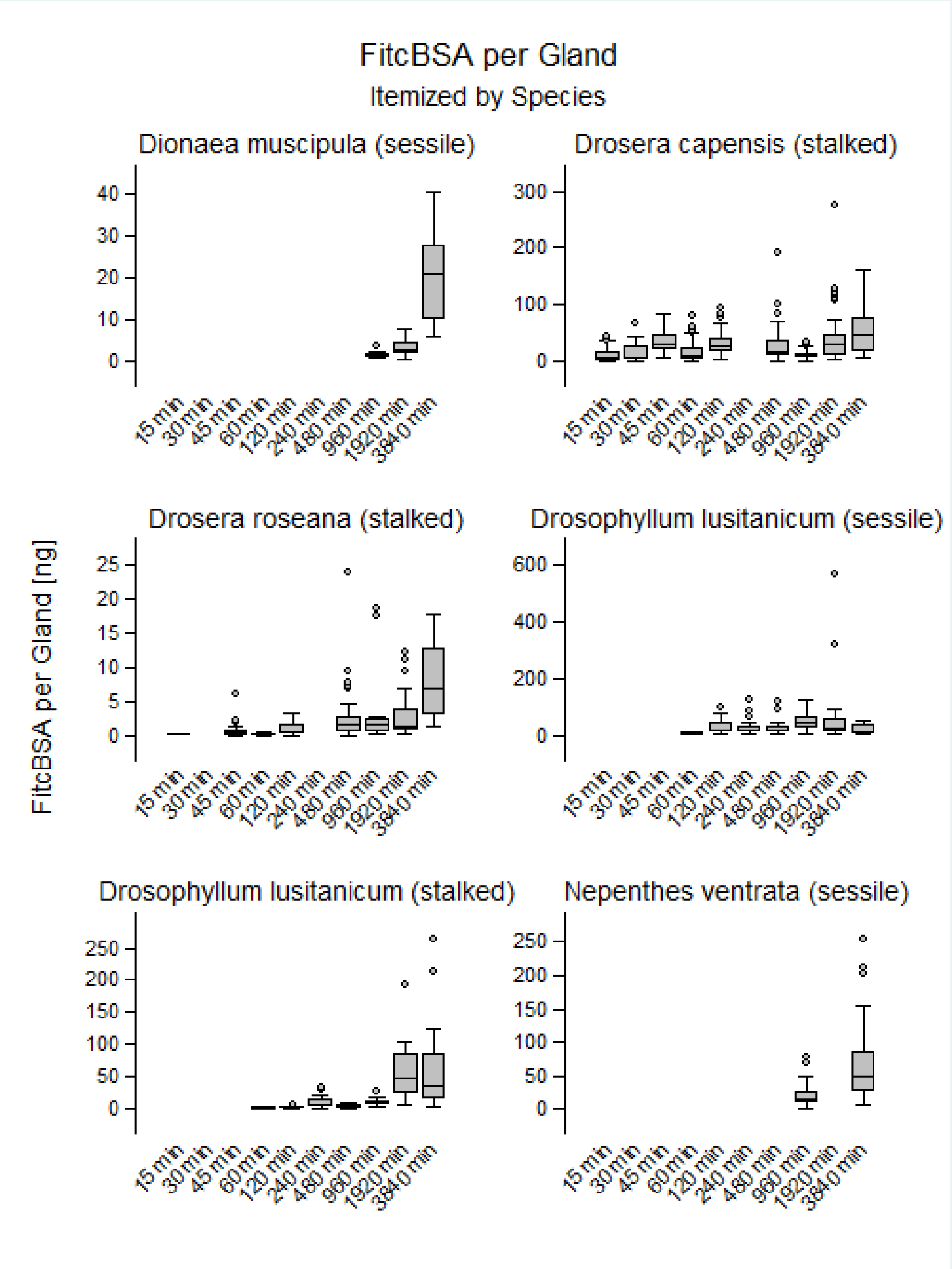

Figure S5. Boxplot of FITC-BSA content in observed glands in *Dionaea muscipula*, *Drosera roseana*, *Drosera capensis*, *Nepenthes* × *ventrata* and stalked and sessile glands in *Drosophyllum lusitanicum*.

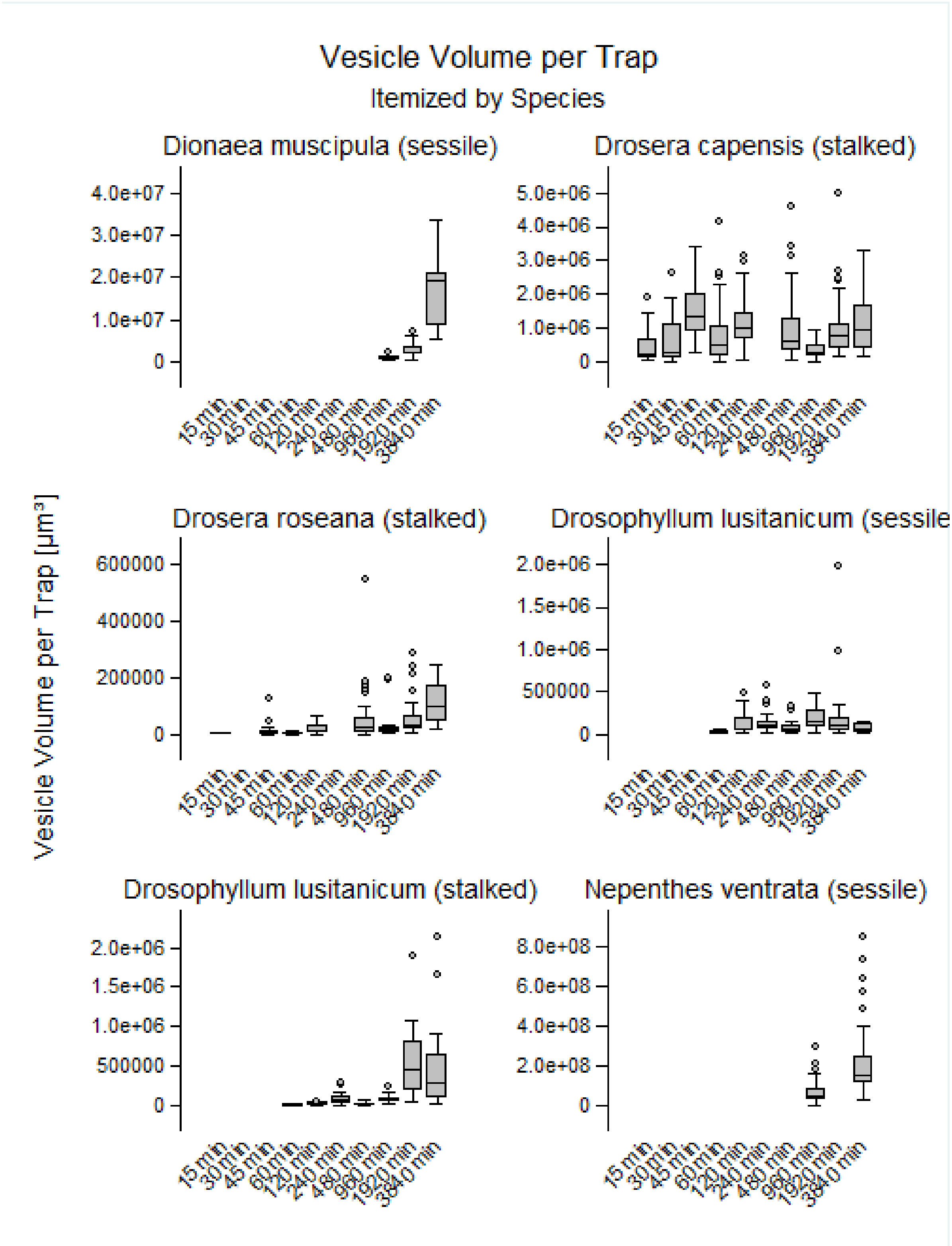

Figure S6. Boxplot of vesicle volume per trap (DZ) in *Dionaea muscipula*, *Drosera roseana*, *Drosera capensis*, *Nepenthes* × *ventrata* and stalked and sessile glands in *Drosophyllum lusitanicum*.

Table S1. Regression analysis itemized by species.

Species	R^2	p-value (Model)	Slope (95% CI)	p-value (Time)	Constant (95% CI)	p-value (Constant)
**Vesicle Number**						
Dionaea muscipula	0,37	< 0.001	-.35 (-.48 - -.21)	< 0.001	41.2 (35.1 - 47.3)	< 0.001
Drosera capensis	0	0,691	-.011 (-.067 - .045)	0,691	10.4 (9.56 - 11.3)	< 0.001
Drosera roseana	0,07	< 0.001	-.041 (-.063 - -.018)	< 0.001	3.94 (3.44 - 4.44)	< 0.001
Drosophyllum lusitanicum (sessile)	0,07	0,001	-.074 (-.12 - -.032)	0,001	9.22 (8.11 - 10.3)	< 0.001
Drosophyllum lusitanicum (stalked)	0,04	0,019	-.057 (-.11 - -.0097)	0,019	12.5 (10.9 - 14.2)	< 0.001
Nepenthes x ventrata	0,05	0,041	-.016 (-.032 - -.00071)	0,041	3.45 (2.68 - 4.22)	< 0.001
**Diameter**						
Dionaea muscipula	0,43	< 0.001	.027 (.026 - .029)	< 0.001	.13 (.061 - .2)	< 0.001
Drosera capensis	0	0,777	-.00018 (-.0014 - .001)	0,777	1.24 (1.21 - 1.26)	< 0.001
Drosera roseana	0,17	< 0.001	.023 (.019 - .027)	< 0.001	.990 (.91 - 1.07)	< 0.001
Drosophyllum lusitanicum (sessile)	0,03	< 0.001	.0095 (.0065 - .012)	< 0.001	1.46 (1.4 - 1.52)	< 0.001
Drosophyllum lusitanicum (stalked)	0,21	< 0.001	.027 (.024 - .03)	< 0.001	1.3 (1.22 - 1.39)	< 0.001
Nepenthes x ventrata	0,17	< 0.001	.025 (.018 - .032)	< 0.001	1.46 (1.12 - 1.8)	< 0.001
**Brightness**						
Dionaea muscipula	0,1	< 0.001	-.2 (-.24 - -.17)	< 0.001	35.7 (34.3 - 37)	< 0.001
Drosera capensis	0,48	< 0.001	1 (.970 - 1.04)	< 0.001	15.8 (15.2 - 16.4)	< 0.001
Drosera roseana	0,19	< 0.001	.49 (.40 - .57)	< 0.001	13.3 (11.7 - 14.9)	< 0.001
Drosophyllum lusitanicum (sessile)	0,1	< 0.001	.21 (.18 - .24)	< 0.001	16.1 (15.5 - 16.8)	< 0.001
Drosophyllum lusitanicum (stalked)	0,09	< 0.001	.12 (.099 - .14)	< 0.001	13.2 (12.6 - 13.9)	< 0.001
Nepenthes x ventrata	0,01	0,111	.053 (-.012 - .12)	0,111	25.9 (22.9 - 29)	< 0.001
**Concentration**						
Dionaea muscipula	0,31	< 0.001	-.035 (-.038 - -.032)	< 0.001	8.29 (8.17 - 8.40)	< 0.001
Drosera capensis	0,48	< 0.001	.08 (.077 - .083)	< 0.001	5.52 (5.47 - 5.56)	< 0.001
Drosera roseana	0,14	< 0.001	.034 (.027 - .041)	< 0.001	5.37 (5.24 - 5.5)	< 0.001
Drosophyllum lusitanicum (sessile)	0,09	< 0.001	.021 (.018 - .025)	< 0.001	5.41 (5.34 - 5.48)	< 0.001

Drosophyllum lusitanicum (stalked)	0,02	< 0.001	.0067 (.0043 - .009)	< 0.001	5.09 (5.02 - 5.16)	< 0.001
Nepenthes x ventrata	0,01	0,066	-.0062 (-.013 - .00041)	0,066	6.56 (6.24 - 6.87)	< 0.001
**Vesicle Volume per Gland**						
Dionaea muscipula	0,66	< 0.001	2.82 (2.23 - 3.41)	< 0.001	-54.6 (-81.7 - -27.5)	< 0.001
Drosera capensis	0	0,522	.044 (-.091 - .18)	0,522	18.9 (16.7 - 21)	< 0.001
Drosera roseana	0,11	< 0.001	.34 (.19 - .49)	< 0.001	5.41 (2.08 - 8.74)	0,002
Drosophyllum lusitanicum (sessile)	0	0,931	.017 (-.38 - .41)	0,931	35.2 (24.9 - 45.6)	< 0.001
Drosophyllum lusitanicum (stalked)	0,22	< 0.001	3.71 (2.46 - 4.95)	< 0.001	26.3 (-16.6 - 69.2)	0,227
Nepenthes x ventrata	0,21	< 0.001	.990 (.57 - 1.41)	< 0.001	6.1 (-14.8 - 27)	0,564
**FITC-BSA Content per Gland**						
Dionaea muscipula	0,63	< 0.001	.018 (.014 - .021)	< 0.001	-.34 (-.52 - -.16)	< 0.001
Drosera capensis	0,07	< 0.001	.0027 (.0017 - .0037)	< 0.001	.1 (.083 - .12)	< 0.001
Drosera roseana	0,17	< 0.001	.0025 (.0016 - .0034)	< 0.001	.025 (.0058 - .045)	0,011
Drosophyllum lusitanicum (sessile)	0	0,485	.00083 (-.0015 - .0032)	0,485	.18 (.12 - .24)	< 0.001
Drosophyllum lusitanicum (stalked)	0,25	< 0.001	.019 (.013 - .025)	< 0.001	.098 (-.1 - .3)	0,332
Nepenthes x ventrata	0,2	< 0.001	.0054 (.0031 - .0078)	< 0.001	.045 (-.074 - .16)	0,454
**Vesicle Volume per Trap**						
Dionaea muscipula	0,66	< 0.001	357000 (282000 - 432000)	< 0.001	-6920000 (-10400000 - -3480000)	< 0.001
Drosera capensis	0	0,522	2070 (-4280 - 8410)	0,522	884000 (783000 - 986000)	< 0.001
Drosera roseana	0,11	< 0.001	1280 (718 - 1850)	< 0.001	20700 (7940 - 33400)	0,002
Drosophyllum lusitanicum (sessile)	0	0,931	64.7 (-1400 - 1530)	0,931	131000 (92200 - 169000)	< 0.001
Drosophyllum lusitanicum (stalked)	0,22	< 0.001	7010 (4650 - 9370)	< 0.001	49800 (-31400 - 131000)	0,227
Nepenthes x ventrata	0,21	< 0.001	3110000 (1790000 - 4420000)	< 0.001	19100000 (-46400000 - 84600000)	0,564
**FITC-BSA Content per Trap**						
Dionaea muscipula	0,63	< 0.001	2230 (1730 - 2720)	< 0.001	-42700 (-65300 - -20000)	< 0.001
Drosera capensis	0,07	< 0.001	127 (78.8 - 175)	< 0.001	4670 (3900 - 5440)	< 0.001
Drosera roseana	0,17	< 0.001	9.61 (6.29 - 12.9)	< 0.001	96.7 (22.2 - 171)	0,011
Drosophyllum lusitanicum	0	0,485	3.1	0,485	671	< 0.001

(sessile)			(-5.64 - 11.8)		(441 - 901)	
Drosophyllum lusitanicum (stalked)	0,25	< 0.001	36.1 (25.2 - 47)	< 0.001	185 (-191 - 560)	0,332
Nepenthes x ventrata	0,2	< 0.001	17000 (9600 - 24500)	< 0.001	140000 (-231000 - 511000)	0,454
